# Tailoring the Microstructure and Mechanical Properties of Phenolic Aerogels with Graphene Oxide

**DOI:** 10.3390/gels12010034

**Published:** 2025-12-30

**Authors:** Congyan Hu, Lei Chen, Zixuan Lei, Yafei Li, Liwei Wang, Yiming Yang, Tong Zhao, Hao Li

**Affiliations:** 1 Key Laboratory of Science and Technology on High-Tech Polymer Materials, Institute of Chemistry, Chinese Academy of Sciences, Beijing 100190, China; hucongyan@iccas.ac.cn (C.H.); chenlei@iccas.ac.cn (L.C.); leizixuan0207@iccas.ac.cn (Z.L.); liyafei9876@163.com (Y.L.); wangliwei@iccas.ac.cn (L.W.); tzhao@iccas.ac.cn (T.Z.); 2 University of Chinese Academy of Sciences, Beijing 100049, China; 3 Beijing National Laboratory for Molecular Sciences, Beijing 100084, China

**Keywords:** phenolic aerogels, graphene oxide, finite-element analysis, mechanical properties, thermal insulation

## Abstract

Phenolic aerogels offer low thermal conductivity, excellent thermal stability, and high char yield, but they suffer from intrinsic brittleness, low compressive modulus, and limited compressive strain. To overcome these limitations, phenolic aerogels modified with graphene oxide were synthesized and their structural, mechanical, and thermal insulation properties were evaluated. The GO fillers were uniformly dispersed in the phenolic matrix without disrupting its porous structure. Mechanical testing revealed that the modified aerogel achieved a compressive modulus of 265.52 MPa, representing a 67% increase over the pure phenolic aerogel’s value of 158.49 MPa, and a compressive strength of 40.19 MPa, compared to 6.18 MPa, for the pure sample. At the same time, the composite maintained good thermal insulation performance, with a thermal conductivity of 0.063 W·m^−1^·K^−1^. This work demonstrates a feasible approach to tailoring the structure–property relationship of phenolic aerogels via GO modification, supporting their potential use in high-temperature insulation and lightweight structural applications.

## 1. Introduction

Phenolic aerogels have emerged as a cornerstone thermal insulation material for extreme environments, leveraging their low thermal conductivity, exceptional thermal stability, and high char yield [1,2,3,4,5,6]. Their application potential is also rapidly expanding into areas such as firesafe building insulation and thermal protection for industrial equipment [7,8,9]. These scenarios, however, impose stringent demands beyond superior insulation, requiring the material to possess sufficient mechanical robustness to withstand operational dynamic loads, such as aerodynamic pressure and installation impacts. A significant bottleneck for pure phenolic aerogels lies in their intrinsic brittleness. Their typical “loose pearl-necklace” skeletal architecture often results in a low compressive modulus and compressive strain [10,11,12]. This inherent structural weakness also manifests during ambient-pressure drying, where the framework is susceptible to powerful capillary forces, leading to significant volume shrinkage, potential pore collapse, and compromised thermal insulation [13,14].

To enhance the mechanical properties of phenolic aerogels, a number of reinforcement strategies have been explored. For instance, Hasegawa et al. synthesized RF aerogels using the amphiphilic block copolymer Pluronic F127, which achieved a compressive modulus of 40 MPa and a compressive strength of 2.7 MPa [15]. However, the process requires solvent exchange and supercritical CO_2_ drying, limiting its large-scale application. Huang et al. fabricated flexible phenolic aerogels backboned by a silicone network through a one-pot synthesis, demonstrating high cyclic compressibility up to 70% strain and good fatigue resistance over 1000 cycles at 40% strain, with a compressive strength of less than 0.5 MPa [16]. But the stepwise curing process requires a high temperature of 180 °C and an autoclave, resulting in an energy-intensive preparation route. Introducing carbon or quartz fibers can improve mechanical properties, but it necessitates complex preforming processes. Despite this approach, the resulting mechanical performance remains relatively low and is still insufficient for high-demand applications [17,18,19]. Therefore, developing a simple and scalable preparation method that can simultaneously confer high modulus and toughness to phenolic aerogels remains a significant challenge.

In contrast, graphene oxide (GO) has demonstrated advantages in polymer systems. Its thin 2D structure and high stiffness, combined with its excellent dispersibility in various solvents, such as water and alcohols, facilitate uniform integration into polymer matrices. Furthermore, the abundant oxygenated functional groups on its surface enable a reinforcement strategy based on forming interfacial bonds [20]. Reports have confirmed that incorporating GO into phenolic resin effectively enhances their mechanical strength and modulus, and improves thermal stability and residual char yield [21,22,23,24,25]. For example, Zhou et al. [22] demonstrated that the introduction of GO significantly enhances the mechanical properties of dense phenolic resin composites. With only 0.5 wt% GO addition, Young’s modulus and tensile strength increased by 22.6% and 39.2%. This provides a theoretical basis and technical reference for leveraging GO to reinforce phenolic aerogels. However, reports on GO-reinforced phenolic aerogels remain relatively scarce. Khalaj et al. conducted a representative study [26]; they prepared novolac/GO aerogels via sol–gel polymerization with ambient-pressure drying. The apparent density reached 0.236 g/cm^3^, the compressive modulus was 25.37 MPa, and the compressive strength was 8.41 MPa. While such studies confirm the feasibility of GO modification, they typically pursue higher GO loadings and often overlook the critical microstructural evolution occurring at low GO concentrations. The relationship between subtle morphological changes, including the degree of GO encapsulation by phenolic nanoparticles, the transition from wrapped to exposed GO sheets, and the onset of GO aggregation, and the resulting macroscopic mechanical properties has not been systematically reported. In addition, there remains a lack of multiscale understanding of reinforcement mechanisms in phenolic aerogels modified with GO. This mechanistic insight is crucial, as evidenced in other complex systems where nanomaterial dispersion and matrix interactions directly govern structural enhancement and performance [27].

To address these gaps, this study employs GO with few layers as a reinforcement additives and systematically investigates its effect across a concentration range of 0.00 to 1.00 wt% on phenolic aerogels. Unlike most prior works that focused on increasing GO content, we demonstrate that an optimal trace amount of GO (0.25 wt%) can induce a unique hybrid microstructure in which GO nanosheets are uniformly wrapped by phenolic nanoparticles. The aerogels were prepared using an ambient-pressure-drying process, without solvent exchange or the use of supercritical CO_2_ or freeze-drying. Concurrently, this work systematically identifies the optimal GO loading from a multiscale perspective and elucidates its reinforcement mechanism, through analyses encompassing GO’s nanoscale architecture, molecular-level covalent bonding, the composite’s microstructural evolution, and the resultant macroscopic properties. The approach has yielded good mechanical properties, with compressive strength and modulus of 40.19 MPa and 265.52 MPa. This straightforward preparation method strengthens the viability of aerogels for thermal insulation applications in harsh environments.

## 2. Results and Discussion

### 2.1. Morphological and Crystalline Structure Characterization of GO

**Figure 1:**
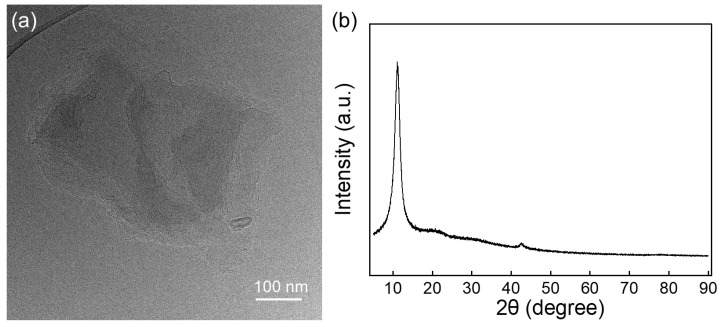
(**a**) TEM image of GO. (**b**) XRD pattern of GO, with the characteristic peak at ~11.09° corresponding to the (001) crystal plane.

The morphological and structural characteristics of GO were analyzed using transmission electron microscopy (TEM) and X-ray diffraction (XRD). As observed in the TEM image of Figure 1a, the micrograph reveals GO as thin, translucent nanosheets with a lateral dimension of approximately 200–300 nm, and the wrinkled and folded edges of the nanosheets are prominent. Studies have suggested that the formation of such slight wrinkles and curled edges can be attributed to the high surface energy of thin GO nanosheets and the introduced oxygen-containing functional groups [28].

The XRD spectra (Figure 1b), measured over a 2θ range of 5° to 90°, show a (001) diffraction peak at 2θ = 11.09° for GO, which corresponds to the interlayer distance between graphene sheets. The interlayer distance (d) between graphene layers, estimated by applying Bragg’s law to the (001) reflection diffraction, is summarized in Table 1. The interlayer spacing of GO was calculated to be 0.80 nm. This value is consistent with the typical interlayer spacing range of GO with few layers, as reported in the characterization of pure GO [29,30]. Using Scherrer’s equation, with a shape factor of K = 0.9, applied to the (001) diffraction peak, the average stacking height of GO was determined to be 4.94 nm. The average number of stacked layers was estimated as 6.2 [31]. This result indicates that GO exhibited a well-exfoliated few-layer structure, which is favorable for its dispersibility and interfacial interactions in composite materials.

(1) $$D=\frac{K\gamma }{Bcos\theta }$$

### 2.2. Preparation and Characterization of GPF

**Figure 2:**
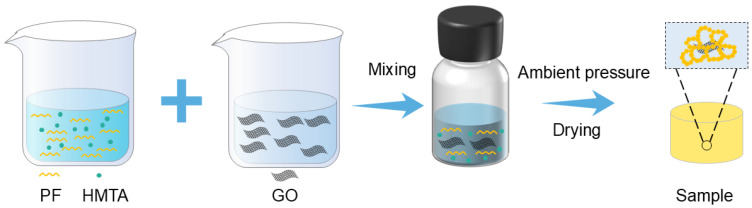
Schematic illustration of the GPF aerogel fabrication process: HMTA, PF, and GO are mixed in solution, followed by ambient-pressure drying to yield the final GPF aerogels.

**Figure 3:**
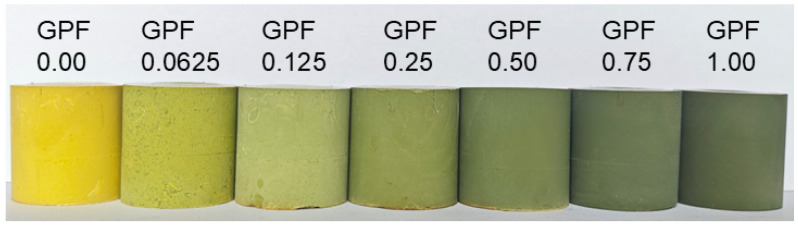
Pictures of GPF aerogels with GO contents ranging from 0.00 to 1.00 wt% relative to the PF, displaying a color gradient from pale yellow to dark green as the GO loading increases from left to right.

The GPF aerogels were fabricated with GO contents of 0.00, 0.0625, 0.125, 0.25, 0.50, 0.75, and 1.00 wt% relative to phenolic formaldehyde matrix. The aerogels were denoted as GPF X. Here, G stands for GO, PF represents phenolic formaldehyde resin, and X indicates the weight percentage of GO relative to PF. PF is referred to as GPF 0.00 in the sample naming. The fabrication process involved a sol preparation, sol–gel transition, and ambient pressure. Commercial GO was purchased as an aqueous dispersion that was already dispersed well in water. First, the required mass of GO was calculated in advance, then a corresponding mass of this GO aqueous dispersion was weighed out, and a certain amount of ethylene glycol was added to it. The mixture was then transferred to a vacuum distillation apparatus. Vacuum rotary evaporation was carried out at 90 °C for 30 min. This process removed water while retaining GO in the ethylene glycol phase, resulting in a stable GO/ethylene glycol dispersion. As illustrated in Figure 2, PF, hexamethylenetetramine (HMTA), deionized water, and additional ethylene glycol were added to the GO/ethylene glycol dispersion. The mixture was stirred continuously for 3 h, resulting in a homogeneous solution. This solution was transferred to a reaction flask and subjected to gelation and aging at 110 °C for 24 h. After gelation, the resulting wet monolithic gels were first dried in air at room temperature for one day. They were then further dried to remove residual solvents through a gradient heating process: 8 h at 80 °C, followed by 8 h at 110 °C, and, finally, 8 h at 130 °C. The obtained aerogels exhibited a distinct color gradient from pale yellow to dark green (Figure 3), with samples ordered from left to right corresponding to GPF 0.00, GPF 0.0625, GPF 0.125, GPF 0.25, GPF 0.50, GPF 0.75, and GPF 1.00. Detailed compositions of each sample are listed in Table 2. The aerogel monoliths exhibited dimensions with a length of 20.955 mm and a diameter of 24.24 mm, where the deviations were 0.34004 mm and 0.20957 mm. The preparation of GPF was facile without sophisticated steps such as high curing temperature, solvent exchange, or supercritical drying, and the resulted aerogel monoliths exhibited almost zero shrinkage without crack, indicating the success of the preparation.

**Figure 4:**
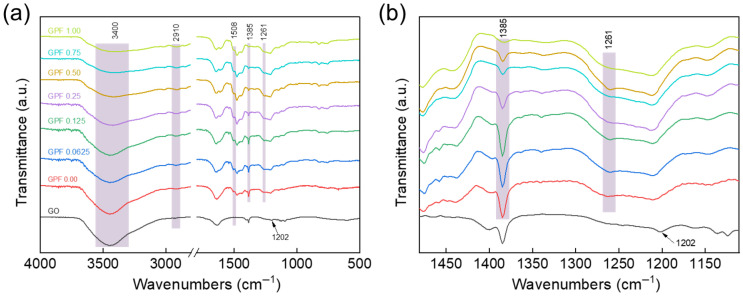
(**a**) FTIR spectra of GO and GPF composites with varying GO loadings. (**b**) Magnified view of the 1100–1480 cm^−1^ region from (**a**).

Fourier-transform infrared (FTIR) spectroscopy was employed to probe the chemical interactions between GO and the PF (Figure 4), with spectra collected over 4000–500 cm^−1^ to characterize key functional groups involved. A broad hydroxyl band (3400 cm^−1^) was observed across all samples, corresponding to residual -OH on GO and phenolic hydroxyls in the resin. In GPF 0.00, this band appeared relatively narrow, whereas in GO loading samples, it exhibited significant broadening and a slight shift to lower wavenumbers. This indicates intermolecular hydrogen bonds between GO’s hydroxyl as well as carboxyl groups and phenolic hydroxyls, weakening -OH covalent bonds and creating a dispersed distribution of -OH vibration frequencies [32]. The weak absorption at 2910 cm^−1^ is attributed to the stretching vibration of C-H bonds [11], while the characteristic peaks at 1508 cm^−1^ correspond to the skeletal vibration of aromatic C=C bonds [33]. For better comparison of spectra, the curves were normalized according to the intensity of the peak at 1508 cm^−1^. Notably, the characteristic peak at 1261 cm^−1^, which is assigned to the stretching vibration of aromatic ether bonds (Ph-O-C), gradually intensifies as the GO loading increases in the range of 0.0625 to 1.00 wt% [34], concurrently with a slight diminishment of the phenolic hydroxyl (Ph-OH) peak at 1385 cm^−1^ [35]. Comparative analysis of the spectra for GO and GPF 0.00 reveals that the epoxy groups on GO (1202 cm^−1^) [36] may undergo a ring-opening reaction. The phenolic hydroxyls (Ph-OH) in the resin act as nucleophiles, attacking the electrophilic carbon atoms of GO’s epoxy groups to form covalent aromatic ether linkages (Ph-O-C).

**Figure 5:**
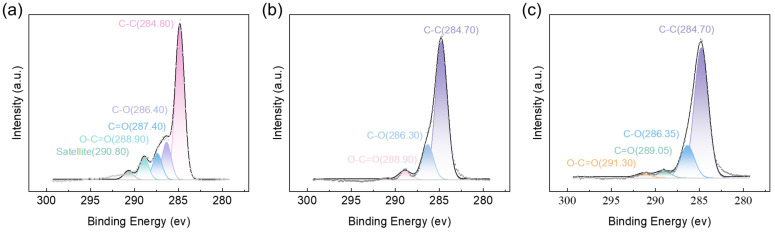
C1s XPS spectra of (**a**) GO, (**b**) GPF 0.00, and (**c**) GPF 1.00.

XPS C1s peaks were used to determine the carbon chemical states of GO, GPF 0.00, and GPF 1.00, with their high resolution spectra shown in Figure 5a–c: Figure 5a presents GO’s C1s spectrum. The C1s spectrum showed peaks at 284.8 eV (C-C), 286.4 eV (C-O), 287.4 eV (C=O), 288.9 eV (O-C=O), and 290.8 eV (π-π* satellite bonds) [37]. Figure 5b displays the C1s spectrum of GPF 0.00, with dominant peaks at 284.7 eV (C-C), 286.3 eV (C-O), and 288.9 eV (O-C=O) [38,39]. Figure 5c shows GPF 1.00’s peaks at 284.70 eV (C-C), 286.35 eV (C-O), 289.05 eV (C=O), and 291.30 eV (O-C=O). Meanwhile, the FWHM of C-O peak of GPF 0.00 has a FWHM of 1.57 eV, while the C-O related peak of GPF 1.00, which includes unreacted Ph-OH groups in the resin, unreacted C-O groups in GO, and potentially newly formed Ph-O-C bonds, shows an increased FWHM of 1.86 eV. According to the mechanism reported in relevant studies, the broader FWHM in GPF 1.00 originates from the coexistence of multiple carbon oxygen chemical states, leading to the superposition of unresolved electronic states, this observation could imply that the interface might potentially involve chemical reactions rather than simple physical mixing. It should be noted that the complete XPS C1s data for samples ranging from GPF 0.00 to GPF 1.00 are summarized in the Supplementary Material.

### 2.3. Morphological and Microstructural Characterization of GPF Composites

**Figure 6:**
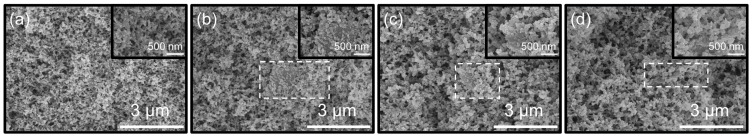
(**a**–**d**) SEM micrographs of GPF composites with GO mass fractions of (**a**) 0.00, (**b**) 0.0625, (**c**) 0.125, and (**d**) 0.25.

**Figure 7:**
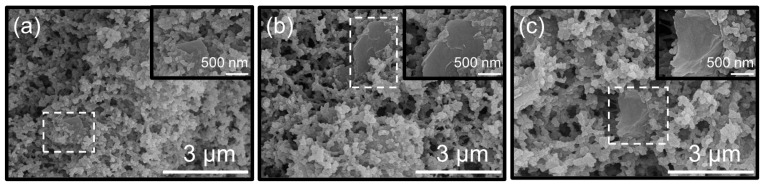
(**a**–**c**) SEM micrographs of GPF composites with GO mass fractions of (**a**) 0.50, (**b**) 0.75, and (**c**) 1.00.

SEM observations in Figure 6a–d and Figure 7a–c reveal a distinct morphological evolution of GPF with increasing GO content. In the GPF 0.00, SEM showed a morphology of porous framework formed by stacking of nanoparticles with an average size of 37 nm (Table 3). From Figure 6b–d, the incorporation of GO induces an increase in the nanoparticle size, while concurrently facilitating their crosslinking and chemical reactions on the GO surface. As GO loading increases moderately across the range of GPF 0.0625 to GPF 0.25, it can be visualized by the white dashed contours and magnified insets that nanoparticles tightly warp GO nanosheets, and the nanoparticle size increases slightly from 42 nm to 54 nm.

However, at higher loadings (GPF 0.50–1.00, Figure 7a–c), the nanoparticle size begins to increase significantly with increasing GO content; meanwhile, the stacking mode of GO and nanoparticles undergoes a substantial change, nanoparticles cannot fully warp the GO, and GO itself also exhibits stacking. As visualized in the top-right inset of Figure 7c (GPF 1.00), GO sheets stack into distinct flaky aggregates, and these macroscopic clusters act as irregular growth centers. These agglomerates locally accelerate phenolic particle growth, creating pronounced heterogeneous regions. Dense particle clusters accumulate in certain regions around GO aggregates, whereas in adjacent areas, the particle population is sufficiently sparse to cause incomplete encapsulation of GO, leaving portions of the nanosheets exposed, and the size of phenolic particles increases to 130 nm. Such a heterogeneous structure impairs the continuity of the porous framework and may give rise to new stress concentration points.

**Figure 8:**
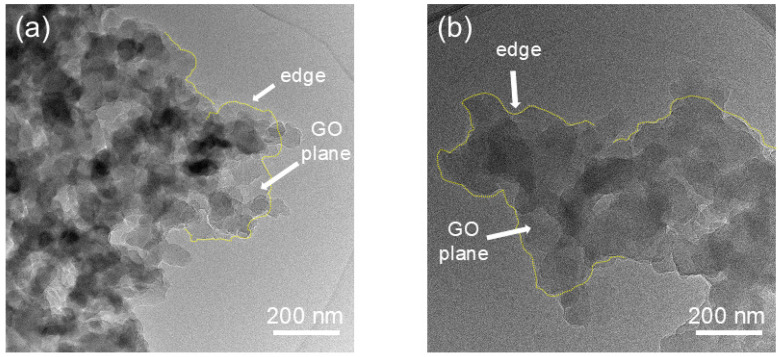
TEM images of GPF composites: (**a**) GPF 0.25 and (**b**) GPF 1.00. Yellow dashed outlines mark the distribution of GO nanosheets within the phenolic matrix.

To visualize the microstructural changes driven by GO incorporation, transmission electron microscopy observations were conducted on sample GPF 0.25, as shown in Figure 8a, and on GPF 1.00, as shown in Figure 8b. In Figure 8a, the yellow dashed outline delineates a GO nanosheet with a thin edge and well-defined contour. Phenolic particles encapsulate its surface. In the case of GPF 1.00, presented in Figure 8b, the GO nanosheet also exhibits a thin edge, but it is only partially coated by phenolic particles. Moreover, a clear increase in phenolic particle size with rising GO content is evident, which is consistent with the trends observed via SEM.

### 2.4. Pore Structure and Porosity Characteristics of GPF Composites

**Figure 9:**
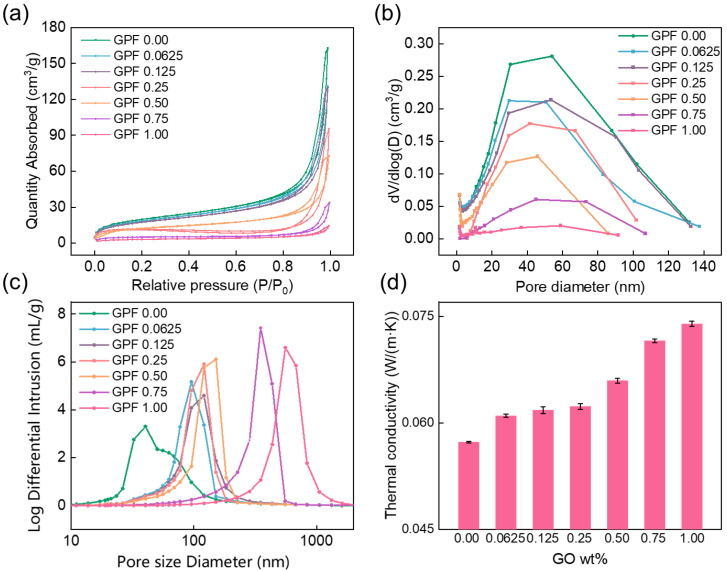
(**a**) N_2_ adsorption–desorption isotherms, (**b**) BJH-derived mesopore size distribution, (**c**) pore size distribution of GPF composites from MIP, and (**d**) thermal conductivity of GPF composites.

The pore architecture of GPF aerogels was systematically probed using N_2_ adsorption–desorption (BET) and mercury intrusion porosimetry (MIP), with the resulting data on surface area, average pore diameter, and porosity summarized in Table 4. All GPF samples exhibit Type IV N_2_ adsorption–desorption isotherms (Figure 9a). These isotherms are characterized by a steep N_2_ uptake at P/P_0_ (0.7–1.0) and a distinct hysteresis loop, both hallmarks of mesopore-driven capillary condensation in the 2–50 nm range. Furthermore, the isotherms show a monotonic decrease in N_2_ uptake with increasing GO content. This decline reflects reduced accessible pore volume and surface area, as Table 4 demonstrates, with values decreasing from 72.38 m^2^/g (GPF 0.00) to 11.56 m^2^/g (GPF 1.00). The BJH plot (Figure 9b) further reveals that the GPF 0.00 has a dominant mesopore population centered at ~30–50 nm, while mesopore volume (dV/dlogD) decreases sharply with higher GO loading MIP (Figure 9c), and the average pore diameter and porosity complement these findings: average pore diameter increases from 45.45 nm (GPF 0.00) to 495.99 nm (GPF 1.00), and porosity rises from 62.54% to 69.72%. Notably, GPF 0.25 maintains an average pore diameter of 93.15 nm, remaining below 100 nm. For aerogels prioritizing mechanical strength and thermal insulation, a pore size below 100 nm is advantageous: pores smaller than 100 nm avoid stress concentration, as large pores are often regarded as structural defects, and concurrently match the mean free path of air molecules, suppressing gas phase heat conduction [40]. The quantitative evolution of mesopore and macropore volumes is detailed in Table S3 of the Supplementary Material. In summary, the two-dimensional sheet-like structure and chemical interactions of GO make the sol–gel process of phenolic nanoparticles tend to occur on the GO sheets; the growth and stacking of these phenolic polymer nanoparticles form larger interparticle gaps, shifting the pore diameter toward macropores and reducing the BET surface area [41,42].

Thermal conductivity serves as a critical parameter for assessing the thermal insulation performance of aerogels, with a strong dependence on their microstructures. The thermal conductivity of GO-reinforced PF with GO weight fractions ranging from 0.00 to 1.00 wt% is presented in Figure 9d. As observed, the GPF 0.00 exhibited the lowest thermal conductivity of 0.057 W·m^−1^·K^−1^. With increasing GO content, the thermal conductivity showed a gradual upward trend: at 0.25 wt% GO, it rose to 0.063 W·m^−1^·K^−1^; at 1.00 t% GO, it reached 0.073 W·m^−1^·K^−1^.

This increase in thermal conductivity can be attributed to two key factors: first, GO nanosheets, inherently possessing relatively high thermal conductivity, tend to form continuous thermal conduction paths within the aerogel matrix as their content increases, accelerating heat transfer [43]; second, excessive GO loading may disrupt the original porous structure of the aerogel, reducing the tortuosity of heat conduction pathways and weakening the insulating effect of pores. Despite the upward trend, (GPF 0.0625–0.25) shows a mild rise in thermal conductivity, which stands in stark contrast to the marked escalation observed at higher GO loadings (GPF 0.50–1.00). This behavior allows for the simultaneous preservation of the aerogel’s thermal insulation capability and the full exploitation of GO’s mechanical reinforcement effects.

### 2.5. Mechanical Properties of GPF Composites

**Figure 10:**
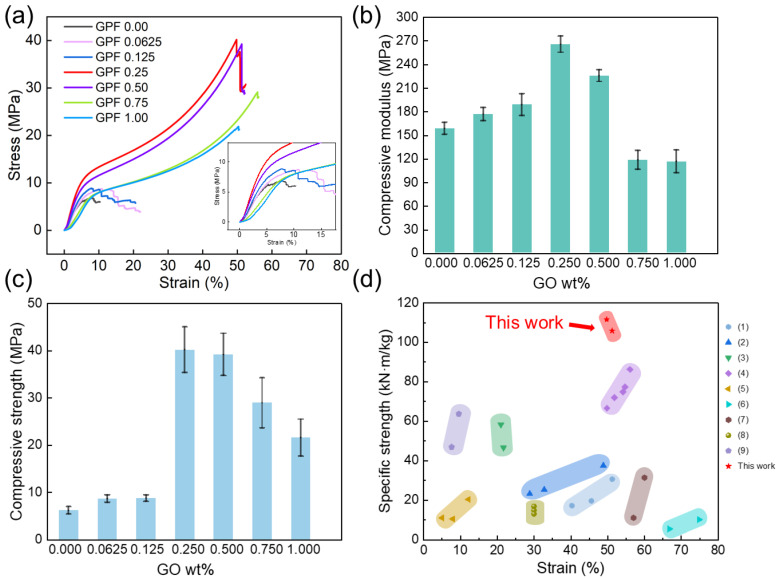
(**a**) Compressive stress–strain curves. (**b**) Compressive modulus. (**c**) Compressive strength. (**d**) Compressive strain and specific compressive strength for various aerogel materials. (1) Phenolic resin aerogel [10]. (2) Boron modified phenolic resin aerogel [11]. (3) Novel phenolic resin/silicone aerogel [44]. (4) Boron modified phenolic aerogel [4]. (5) Zr/Si preceramic polymer-hybridized phenolic resin aerogel [45]. (6) Bifunctional silicone-modified phenolic aerogel [46]. (7) Carbon fiber felt-reinforced resorcinol formaldehyde aerogel [47]. (8) Quartz/carbon hybrid fiber-reinforced silicon-phenolic interpenetrating aerogel [48]. (9) Polysilazane-modified phenolic resin/carbon fiber fabric aerogel [49].

The mechanical properties and thermal conductivity of PF reinforced with GO were systematically analyzed. Compressive stress–strain curves, compressive modulus profiles, and compressive strength are shown in Figure 10. Corresponding specific values, including density, are summarized in Table 5. As shown in the compressive stress–strain curves of Figure 10a, the GPF 0.00 exhibited intrinsic brittleness, presenting a low initial slope and premature fracture within a narrow strain range, with fracture occurring under a load of 6.18 MPa. In contrast, the introduction of GO induced a distinct mechanical evolution: for samples with GO contents of 0.0625 wt% and 0.125 wt%, although the compressive modulus increased, their compressive strength remained relatively low, indicating persistent brittleness comparable to the pure aerogel. At 0.25 wt% GO, the stress–strain curves exhibited simultaneously steeper elastic segments, higher peak stresses, and a significantly extended strain range before fracture, demonstrating synergistic enhancement in strength, and toughness. This is corroborated by the compressive strength data in Figure 10c, which reaches 40.19 MPa. When GO content exceeded 0.25 wt%, as seen in samples with 0.75 wt% and 1.00 wt%, the compressive modulus declined, and the compressive strength gradually decreased. However, the compressive strain was notably wider than that of the low-content GO samples ranging from 0.0625 to 0.125 wt%. This indicates reduced brittleness despite the compromised modulus. The deterioration in modulus at high GO content is attributed to defects induced by agglomeration, while the retained toughness shows the dual nature of GO loading in tailoring mechanical responses. The fracture strain of each sample and breaking energy is summarized in the Supplementary Material. The density of all GPF aerogels remained relatively constant (~0.36–0.38 g/cm^3^), indicating that the mechanical enhancement originated from the structural regulation by GO rather than density variation. A comparison of GPF aerogels with other phenolic aerogels in specific strength and strain (Figure 10d) reveals their superior mechanical properties, suggesting that GPF aerogels are promising candidates for lightweight structural applications.

### 2.6. Conceptual Visualization of Mechanical Reinforcement Mechanisms via Finite-Element Modeling

**Figure 11:**
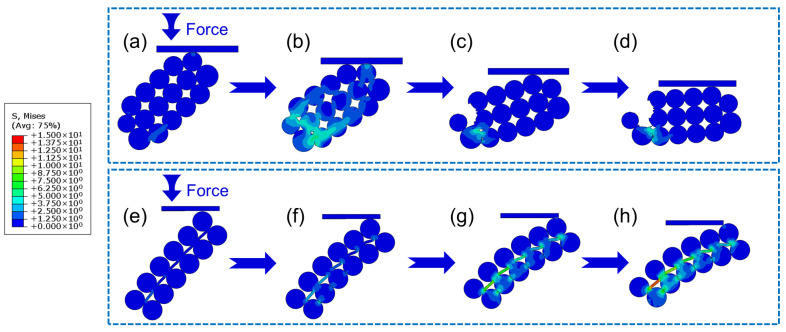
Von Mises stress distribution of GPF 0.00, (**a**–**d**) and GPF 0.25, (**e**–**h**) under mechanical loading.

To address the knowledge gap in the mechanical enhancement mechanism of GO-modified PF, finite-element simulation was conducted via Abaqus 2022 to compare the mechanical behavior of GPF 0.00 (Figure 11a–d) and GPF 0.25 (Figure 11e–h) under loading. The simulation used CPS4R elements, which are four node plane stress quadrilaterals with reduced integration, and adopted explicit dynamics.

Two typical aerogel skeleton models were constructed: GPF 0.00 is composed of stacked nanoparticles, while GPF 0.25 is a composite structure of nanoparticles combined with 2D GO, as shown in Figure 11. The GPF 0.00 model consisted of 928 elements and used an actual modulus of 100 MPa and tensile strength of 7 MPa, while the GPF 0.25 model consisted of 1592 elements and adopted 200 MPa and 40 MPa. Particle adhesion was defined in two ways: particles fully adhered to each other adopted a shared-node configuration with no debonding allowed, while particles not initially adhered had surface-to-surface contact where contact stress develops under compressive loading and separation is possible. Additionally, the GO–PF interface in the models was defined as a bonded shared-node configuration without debonding. At the onset of loading, GPF 0.00 (Figure 11a) exhibited significant local stress concentration at the contact points between spherical particles. GPF 0.25 (Figure 11e), in contrast, displayed more uniform stress propagation across the spherical matrix, with stress gradually distributing along the interconnected PF/GO interfaces. As loading progressed, the relevant results show that GPF 0.00 underwent rapid structural degradation, where initial stress concentrations developed into microcracks, leading to brittle fracture. In contrast, GPF 0.25 maintained structural integrity even at high strain. The model could qualitatively simulate certain failure modes, and the general trends were consistent with the experimental observations.

**Figure 12:**
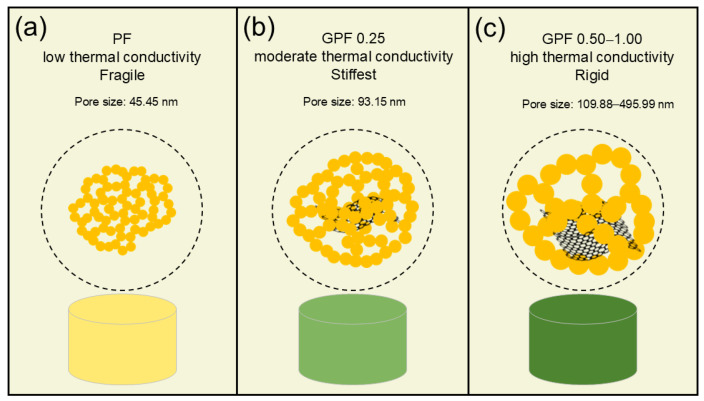
Schematic models depicting phenolic aerogels: (**a**) pure phenolic aerogel, (**b**) PF modified with an optimal GO loading, and (**c**) PF modified with excessive GO.

This behavior can be explained by GO’s dual reinforcement mechanisms [50]: firstly, crack initiation inhibition, where GO nanosheets disperse local stress to suppress microcrack formation at particle interfaces; and, secondly, crack propagation delay, where strong interfacial adhesion between GO and the phenolic matrix resists crack growth, thus enhancing the composite’s ductility. In conclusion, PF has low thermal conductivity but is brittle (Figure 12a). Conversely, PF modified with an optimal GO loading achieves a balance, demonstrating moderate thermal conductivity and the highest stiffness (Figure 12b). However, when modified with excessive GO, the composite shows high thermal conductivity and a rigid mechanical response (Figure 12c).

## 3. Conclusions

In this study, samples of PF containing 0.00 to 1.00 wt% GO were fabricated and systematically characterized. Structural and chemical analyses revealed that moderate GO loadings enable uniform dispersion within the matrix without disrupting its porous architecture, while interfacial interactions enhance adhesion at their interface. Mechanical testing revealed a nonmonotonic trend in compressive properties with GO content. The GPF 0.25 composite achieved a compressive modulus of 265.52 MPa and a compressive strength of 40.19 MPa, while the GPF 0.00 exhibited values of 158.49 MPa and 6.18 MPa, representing a 67% increase in modulus. This enhancement was attributed to synergistic nanoscale reinforcement and improved interfacial compatibility, addressing the porosity–mechanical robustness trade-off. Thermal conductivity characterization further demonstrated a low value of 0.063 W·m^−1^·K^−1^ for GPF 0.25. Overall, this research establishes a feasible strategy for tuning the relationship between structure and performance in phenolic aerogels through GO reinforcement, with GPF 0.25’s enhanced modulus and low thermal conductivity laying a foundation for high-temperature insulation and lightweight structural applications.

## 4. Materials and Methods

### 4.1. Materials

PF was supplied by Shandong Jinan Shengquan Co., Ltd., Jinan, China. HMTA was obtained from Sinopharm Chemical Reagent Co., Ltd. (Shanghai, China). Ethylene glycol was obtained from Tianjin Kangkede Technology Co., Ltd. (Tianjin, China). GO was purchased from Changzhou Sixth Element Material Technology Co., Ltd. (Changzhou, China). Deionized water was used as the solvent in experimental procedures. All reagents were used as received without further purification.

### 4.2. Characterization Techniques

Functional group variations and chemical bonding interactions were analyzed using a Bruker Tensor-27 Fourier-transform infrared spectrometer (Bruker, Bremen, Germany). Aerogel samples were ground into fine powder, mixed with a certain mass of KBr, and pressed into transparent flakes under a pressure of 10 MPa. Spectra were recorded in the wavenumber range of 400–4000 cm^−1^, and 32 scans were accumulated for each sample to reduce noise. XPS measurements were performed using a Thermo Scientific ESCALAB 250XI spectrometer (ThermoFisher, Waltham, MA, USA). The instrument operated with an Al K_α_ radiation source (1486.6 eV), and binding energy was calibrated via the C1s peak at 284.8 eV. XRD measurements were conducted using a Rigaku SmartLab diffractometer (Rigaku Corporation, Tokyo, Japan). The instrument utilized Cu K_α_ radiation (λ = 1.5418 Å), with scans performed over a 2θ range of 5°–90° at a rate of 5 °/min.

TEM was employed for nanoscale morphological characterization using a JEOL JEM-F200 field emission transmission electron microscope (JEOL Ltd., Tokyo, Japan). Bright-field TEM images were captured from multiple regions of each sample using Gatan Microscopy Suite software 3.0, with morphological analysis conducted via the same software. SEM was employed to characterize the porous morphology of GPF aerogels. After fracturing samples to expose fresh cross-sections, they were sputter-coated with a platinum layer for conductivity enhancement. Observations were carried out on a Hitachi SU8020 field emission scanning electron microscope (Hitachi High-Tech Corporation, Tokyo, Japan), and the porous structures as well as particle sizes were analyzed using ImageJ software (ImageJ 1.54g). The micro- and mesoporous structures of the samples were characterized via N_2_ sorption isotherms measured using a Quantachrome Instruments analyzer (Quantachrome Instruments, Boynton Beach, FL, USA). Prior to testing, all samples were subjected to outgassing at 105 °C for 8 h to remove adsorbed impurities. The specific surface area was calculated, applying the BET method, while the BJH approach was used to determine the pore size distribution. MIP measurements were performed using a Micromeritics Autopore V 9620 instrument (Micromeritics Instrument Corporation, Norcross, GA, USA) to characterize the porous structure. This instrument covers a pore size range of 0.003–800 µm and operates under a pressure range of 0.2 psi to 60,000 psi.

Compressive mechanical properties of GPF aerogels were evaluated using an Instron 5567 universal testing machine (Instron, Norwood, MA, USA). Cylindrical samples were compressed at a crosshead speed of 1 mm/min, with stress–strain curves recorded throughout the test. The modulus was determined from the linear region of these stress–strain curves. The thermal conductivity of the samples was measured using a Hot Disk TPS 2500S instrument (Hot Disk Instruments, Uppsala, Sweden).

Mechanical responses of GPF aerogel models were simulated via Abaqus 2022, featuring two structures: (1) monocomponent sphere models (37.5 nm, corresponding to GPF 0.00), and (2) 54 nm sphere models fully encapsulating GO nanosheets (corresponding to GPF 0.25). The lower left boundary was fixed, and a diagonal load was applied until fracture, with real-time monitoring of stress and strain.

The simulation followed the mechanical equilibrium equation:

(2) ∇⋅σ+f=0

where the Cauchy stress tensor (σ) is given by

(3) $$\sigma_{x}=\frac{E}{\left(1+\nu \right)\left(1-2\nu \right)}\;\left[\left(1-\nu \right)\epsilon_{x}+\nu \left(\epsilon_{y}+\epsilon_{z}\right)\right]$$

(4) $$\sigma_{y}=\frac{E}{\left(1+\nu \right)\left(1-2\nu \right)}\;\left[\left(1-\nu \right)\epsilon_{y}+\nu \left(\epsilon_{x}+\epsilon_{z}\right)\right]$$

(5) $$\sigma_{z}=\frac{E}{\left(1+\nu \right)\left(1-2\nu \right)}\;\left[\left(1-\nu \right)\epsilon_{z}+\nu \left(\epsilon_{x}+\epsilon_{y}\right)\right]$$

The relevant parameters are

σ_x_, σ_y_, σ_z_: normal stresses (Pa), ε_x_ ε_y_ ε_z_: normal strains (dimensionless), E: Young’s modulus (Pa), ν: Poisson’s ratio. In this simulation, E is implemented as a function of spatial coordinates, i.e., E(x, y).

The strain components can be written as

(6) $$\varepsilon =\frac{1}{2}\left(\nabla u+{\left(\nabla u\right)}^{T}\right)$$

where u is the displacement tensor.

For perfect elastoplastic materials, the yield surface is fixed in the space of principal stresses; therefore, plastic deformations occur only when the stress path moves on the yield surface.

In the ideal elastic–plastic theory, the stress state inside the material can be characterized in the following way:

(7) $$\sigma =\sigma_{e}\left(\sigma <\sigma_{s}\right)$$

(8) $$\sigma =\sigma_{y}\left(\sigma \geq \sigma_{s}\right)$$

where σ_e_ means the elastic stress computed by the above equations, and σ_s_ means the yield stress. In most cases, it represents the von Mises stress, and the definition for von Mises stress can be written as

(9) $$\bar{\sigma }=\frac{1}{\sqrt{2}}\sqrt{{\left(\sigma_{x}-\sigma_{y}\right)}^{2}+{\left(\sigma_{y}-\sigma_{z}\right)}^{2}+{\left(\sigma_{z}-\sigma_{x}\right)}^{2}+6\left(\tau_{xy}^{2}+\tau_{yz}^{2}+\tau_{zx}^{2}\right)}=\sigma_{s}$$

By combining all the above equations, the stress distribution inside the particles can be obtained.

## Figures and Tables

**Table 1:** XRD characterization results of GO for the (001) diffraction peak.

Sample	2θ (deg)	FWHM (deg)	L_c_ (nm)	d (nm)	n
GO	11.09	1.60	4.94	0.80	6.2

**Table 2:** Compositions of GPF aerogel samples.

Sample	PF (g)	HMTA (g)	EG (g)	H_2_O (g)	GO (g)
GPF 0.00	3.0	0.6	6.6	1.8	0
GPF 0.0625	3.0	0.6	6.6	1.8	2.25 × 10^−3^
GPF 0.125	3.0	0.6	6.6	1.8	4.50 × 10^−3^
GPF 0.25	3.0	0.6	6.6	1.8	9.00 × 10^−3^
GPF 0.50	3.0	0.6	6.6	1.8	1.80 × 10^−2^
GPF 0.75	3.0	0.6	6.6	1.8	2.70 × 10^−2^
GPF 1.00	3.0	0.6	6.6	1.8	3.60 × 10^−2^

**Table 3:** Particle sizes of GPF composites.

Sample	Particle Size (nm)
GPF 0.00	37.45
GPF 0.0625	42.64
GPF 0.125	43.57
GPF 0.25	54.17
GPF 0.50	60.79
GPF 0.75	88.85
GPF 1.00	130.11

**Table 4:** Porosity and textural properties of GPF composites with different GO loadings.

Sample	S_BET_, (m^2^/g)	d_avg_, (nm)	ε (%)	Thermal Conductivity (W·m^−1^·K^−1^)
GPF 0.00	72.38	45.45	62.54	0.057
GPF 0.0625	67.61	64.58	63.26	0.061
GPF 0.125	63.47	69.84	63.47	0.062
GPF 0.25	46.51	93.15	64.20	0.063
GPF 0.50	44.97	109.88	65.51	0.066
GPF 0.75	19.27	263.77	68.09	0.071
GPF 1.00	11.56	495.99	69.72	0.073

S_BET_: Brunauer–Emmett–Teller specific surface area; d_avg_: average pore diameter; ε: porosity.

**Table 5:** Compressive modulus, thermal conductivity, and density of GPF composites with varying GO loadings.

Sample	Compressive Modulus (MPa)	Compressive Strength (MPa)	Density (g/cm^3^)
GPF 0.00	158.49	6.18	0.38
GPF 0.0625	176.59	8.70	0.38
GPF 0.125	188.76	8.77	0.36
GPF 0.25	265.52	40.19	0.36
GPF 0.50	225.48	39.18	0.37
GPF 0.75	118.37	28.95	0.36
GPF 1.00	116.47	21.58	0.37

## Data Availability

The original contributions presented in this study are included in the article/Supplementary Materials. Further inquiries can be directed to the corresponding authors.

## Supplementary Materials

The following supporting information can be downloaded at: https://www.mdpi.com/article/10.3390/gels12010034/s1. **Figure S1**: C1s X-ray photoelectron spectroscopy: (a) graphene oxide and (b–h) GPF composites with GO loadings spanning 0.00 wt% to 1.00 wt%; Figure S2. Compressive stress–strain curves, identifying yield point, ultimate stress, and crack point. Figure S3. Compressive stress-strain profiles of GPF aerogel. Table S1: Binding energy and FWHM of the C–O peak in the C1s XPS spectra. Table S2. Stress and strain values at yield, ultimate stress, and crack points for GPF samples. Table S3. Compressive mechanical properties of GPF aerogels. Table S4. Significance of compressive modulus/strength differences between GPF aerogels. Table S5. Pore volumes of GPF samples measured by BET and MIP techniques.
