# Supplementary material for: Tailoring the Microstructure and Mechanical Properties of Phenolic Aerogels with Graphene Oxide
Source: Gels. 2025 Dec 30;12(1):34. doi: 10.3390/gels12010034 (PMC12840860; doi:10.3390/gels12010034)
Supplement: Supplementary file 1 [file gels-12-00034-s001.zip › gels-4029462-supplementary.pdf]

Supporting Information for

Tailoring the Microstructure and Mechanical Properties of Phenolic Aerogels with Graphene Oxide

Congyan Hu [1,2], Lei Chen [1,2], Zixuan Lei [1,3], Yafei Li [1,2], Liwei Wang [1,2], Yiming Yang [1,2,*], Tong Zhao [1,2,*] and Hao Li [1,2,*].

[1]Key Laboratory of Science and Technology on High-tech Polymer Materials, Institute of Chemistry, Chinese Academy of Sciences, Beijing 100190, China

[2]University of Chinese Academy of Sciences, Beijing 100049, China.

[3]Beijing National Laboratory for Molecular Sciences

[*]Correspondence: yiming924@iccas.ac.cn.

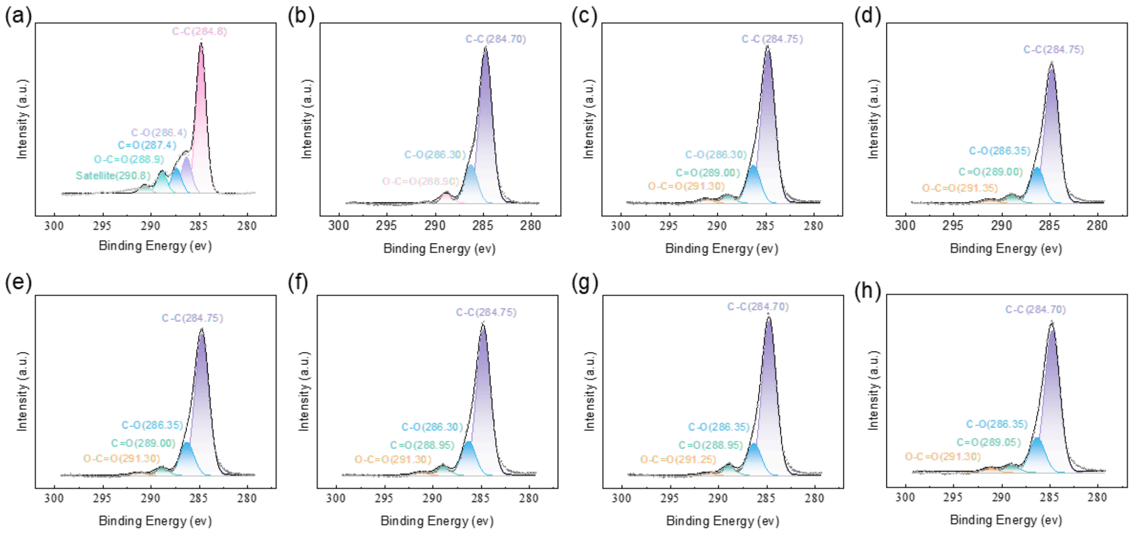

Figure S1. C 1s X-ray photoelectron spectroscopy: (a) graphene oxide, and (b–h) GPF composites with GO loadings spanning 0.00 wt% to 1.00 wt%.

Table S1. Binding Energy and FWHM of the C–O Peak in the C 1s XPS Spectra

Sample	Binding Energy of C-O (eV)	FWHM of C-O (eV)
GPF 0.00	286.30	1.57
GPF 0.0625	286.30	1.67
GPF 0.125	286.35	1.70
GPF 0.25	286.35	1.72
GPF 0.50	286.30	1.73
GPF 0.75	286.35	1.74
GPF 1.00	286.35	1.86

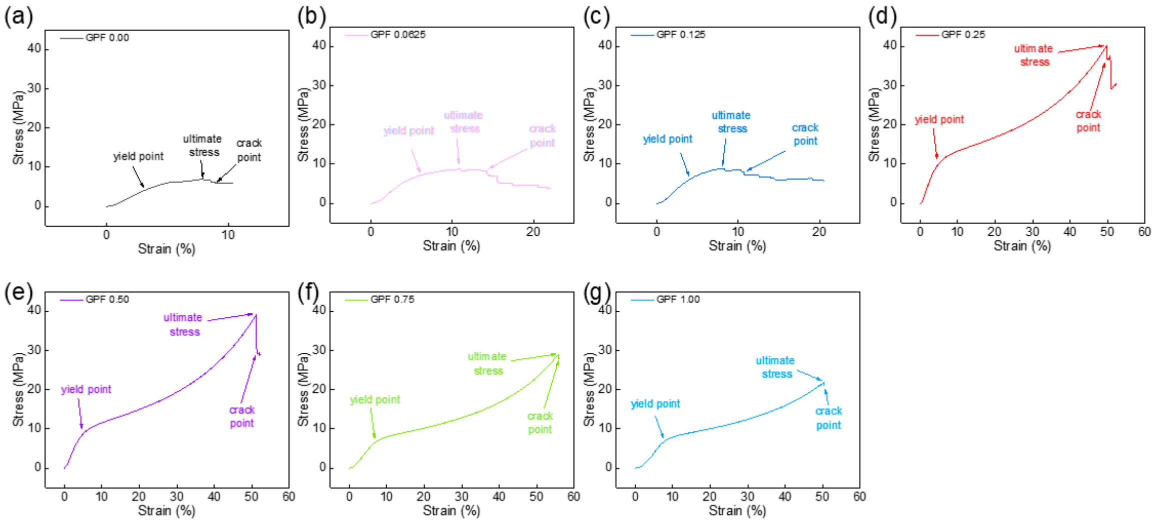

Figure S2. Compressive stress-strain curves, identifying yield point, ultimate stress, and crack point.

Table S2. Stress and strain values at yield, ultimate stress, and crack points for GPF samples.

Sample	Yield Stress (MPa)	Yield Strain (%)	Ultimate Stress (MPa)	Ultimate Strain (%)	Crack Stress (MPa)	Crack Strain (%)	Breaking Energy (eV)
GPF 0.00	4.10	3.12	6.86	7.92	5.95	8.95	41.30
GPF 0.0625	7.16	6.09	8.73	10.80	8.24	14.13	90.31
GPF 0.125	6.15	4.04	8.76	8.00	8.10	10.75	65.72
GPF 0.25	9.96	4.84	40.19	49.76	36.64	49.90	1009.88
GPF 0.50	8.73	4.99	39.24	51.26	29.37	51.39	968.50
GPF 0.75	6.76	7.18	29.10	55.81	27.88	55.93	746.74
GPF 1.00	6.82	7.76	21.82	50.24	21.12	50.33	579.94

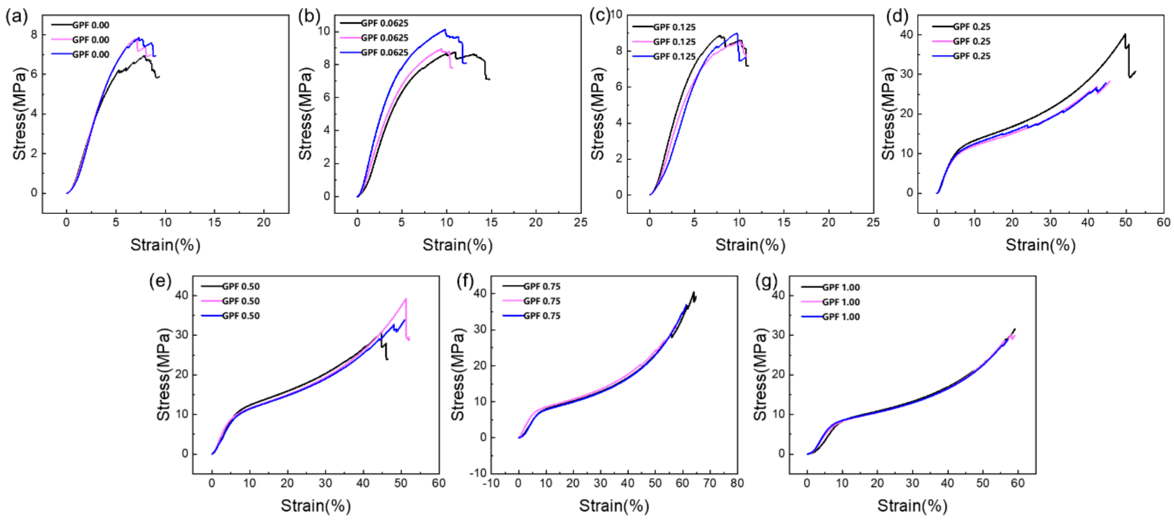

Figure S3. Compressive stress-strain profiles of GPF aerogel

Table S3. Compressive mechanical properties of GPF aerogels

		GPF 0.00	GPF 0.0625	GPF 0.125	GPF 0.25	GPF 0.50	GPF 0.75	GPF 1.00
Compressive Modules(MPa)	1	158.49	176.59	188.76	265.52	225.48	118.37	116.47
	2	163.06	172.79	187.54	276.04	214.90	121.40	128.27
	3	166.91	175.21	165.06	247.74	208.14	126.19	104.06
	Mean±SD	162.82±4.10	174.86±1.92	180.45±12.03	263.10±14.17	216.17±8.77	121.99±3.92	116.27±12.14
Compressive Strength(MPa)	1	6.23	10.10	8.77	39.87	39.04	28.95	21.58
	2	7.86	8.84	8.94	26.61	32.71	27.02	27.33
	3	7.74	8.70	8.36	26.94	30.74	34.84	28.64
	Mean±SD	7.28±0.89	9.21±0.75	8.69±0.30	31.14±7.56	34.16±4.21	30.27±3.93	25.85±3.60

Table S4. Significance of compressive modulus/strength differences between GPF aerogels.

Adjacent Sample Pairs	P-value for Compressive Modulus	Significance (p<0.05) Compressive Modulus	P-value for Compressive Strength	Significance (p<0.05) Compressive Strength
GPF 0.00 vs GPF 0.0625	0.087	Not Significant	0.042	Significant
GPF 0.0625 vs GPF 0.125	0.413	Not Significant	0.865	Not Significant
GPF 0.125 vs GPF 0.25	0.009	Significant	0.003	Significant
GPF 0.25 vs GPF 0.50	0.045	Significant	0.805	Not Significant
GPF 0.50 vs GPF 0.75	0.002	Significant	0.028	Significant
GPF 0.75 vs GPF 1.00	0.891	Not Significant	0.197	Not Significant

Table S5. Pore volumes of GPF samples measured by BET and MIP techniques.

Sample	Pore volume from BET (cm^3/g)	Pore volume from MIP (cm^3/g)
GPF 0.00	0.251	1.51
GPF 0.0625	0.202	1.56
GPF 0.125	0.200	1.57
GPF 0.25	0.147	1.61
GPF 0.50	0.112	1.67
GPF 0.75	0.052	1.78
GPF 1.00	0.022	1.89

Table S3 quantifies the pore volume evolution with GO loading: the volume dominated by mesopores (BET, 2–50 nm) decreases monotonically from 0.251 cm^3/g to 0.022 cm^3/g, while the volume dominated by macropores (MIP, >50 nm) increases from 1.51 cm^3/g to 1.89 cm^3/g. This reciprocal trend confirms the transition from mesopores to macropores induced by GO.
